# Effects of physical activity on blood pressure and mortality among aged hypertensive patients: A cross-sectional study

**DOI:** 10.1097/MD.0000000000040413

**Published:** 2024-11-01

**Authors:** Zhi Zhang, Cheng Xu, Wanqi Yu, Changqing Du, Lijiang Tang, Xiaowei Liu

**Affiliations:** a Department of Cardiology, First People’s Hospital of Linping District, Hangzhou, Zhejiang, P. R. China; b Science and Education Department, Guangxi Zhuang Autonomous Region Jiangbin Hospital, Nanning, Guangxi, P.R. China; c Department of Medical Records and Statistics, Zhejiang Hospital, Hangzhou, Zhejiang, P. R. China; d Department of Cardiology, Zhejiang Hospital, Hangzhou, Zhejiang, P. R. China.

**Keywords:** aged, blood pressure, hypertension, mortality, physical activity

## Abstract

Previous research on physical activity (PA) has mostly concentrated on a single or small number of activities, with scant coverage of the effects of PA on hypertension (HTN) and all-cause mortality. Most studies examining HTN in the elderly have been too small or shown contradictory findings. We conducted a cross-sectional study using 10 cycles of the National Health and Nutrition Examination Survey data from 1999 to 2018. Our sample consisted of respondents aged 65 years or older with HTN, who underwent thorough in-person home interviews. We used a questionnaire to assess their PA levels and divided them into 2 groups: physically active and inactive. We then used logistic analysis to determine the association between PA and death in HTN patients. The gender distribution was nearly equal among the 11,258 participants, with a mean age of 74.36 ± 5.88 years. Nearly 80% of the survey respondents identified as non-Hispanic White. Patients in the physically active group were less likely to suffer from co-morbidities than those in the inactive group. A negative correlation was found between physically active and systolic blood pressure (*P* < .0001) and a positive correlation between physically active and diastolic blood pressure (*P* = .0007). There was a much higher risk of death from any cause and heart disease in the inactive group in the uncorrected COX model (HR 2.96, CI 2.65–3.32, *P* < .0001; HR 3.48, CI 2.64–4.58, *P* < .0001). The risk of death from any cause and HTN mortality was still significantly higher in the physically inactive group, even after controlling for age, sex, and race or taking all covariates into account. These results have the potential to significantly impact healthcare practices, particularly in the field of geriatric care, by emphasizing the importance of PA in reducing the risk of HTN and mortality in aged patients. The present study underscores the significant benefits of PA in patients aged 65 years and older with HTN. Notably, it was found to reduce systolic blood pressure and have a positive impact on the decrease of all-cause and hypertensive mortality. These findings highlight the crucial role of PA in the health and longevity of aged patients with HTN.

## 1. Introduction

More than 1 billion individuals across the globe are affected with hypertension (HTN), a recognized risk factor for cardiovascular disease, which is becoming an increasingly pressing issue in global health.^[1]^ Studies have shown that people with HTN depend significantly on medicine and that to control their blood pressure (BP), they often need to take numerous antihypertensive drugs.^[2–4]^ This, however, not only raises the cost but also causes unsavory side effects.^[5]^ The most recent expense estimate for HTN, including direct and indirect expenses, is USD 400 billion.^[6]^ Not only that, but the prevalence of HTN rises with age as well, reaching almost 70% among the aged.^[7–9]^ Epidemiological studies have also linked uncontrolled HTN to a host of consequences, such as cardiovascular disease and renal failure.^[10]^

One of the leading causes of cardiovascular illness and death globally is HTN.^[1]^ It is a major risk factor for cardiovascular disease in the elderly, which accounts for around 13.5% of all deaths worldwide.^[11]^ The risk of cardiovascular disease is doubled for every 20/10 mm Hg increase in systolic/diastolic blood pressure (SBP/DBP).^[12]^ Cardiovascular events and all-cause mortality are still linked to high SBP levels, even in elderly HTN patients who are taking antihypertensive medication.^[13]^ To reduce the possibilities of cardiovascular events and mortality, it is crucial to focus on reducing BP in elderly individuals with HTN.^[14,15]^

One of the most basic non-drug treatments for HTN is physical activity (PA).^[16]^ The European Society of Cardiology’s 2020 Exercise Cardiology and Exercise Guidelines for People with Cardiovascular Disease stress the significance of exercise as an integral part of HTN therapy. Moderate PA for 30 minutes, 5 to 7 days a week (or at least 7.5 metabolic equivalent hours) is recommended.^[17]^ One session of high-velocity resistance workouts had an antihypertensive impact, according to a study examining the effects on ambulatory BP in hypertensive older women.^[18]^ Researchers have shown that older patients can reduce their BP with just 1 session of power exercise training in the office. However, this impact does not last under ambulatory conditions with HTN.^[19]^ He and colleagues examined 46 participants and concluded that intense exercise causes a significant increase in BP, but brisk walking can mitigate this effect.^[20]^ In a different study, researchers found that HTN in older people can be acutely reduced BP with as little as 1 aerobic exercise session.^[21]^ PA can be classified into 4 types: those dealing with professional employment, those related to all-day sports, those belonging to a household, and those relating to leisure time.^[22]^ Although there are many of these things to do, most prior research has focused solely on a couple of them. Moreover, most current research on aged individuals with HTN is conducted on a limited scale and produces contradictory findings.^[23]^ Furthermore, prior studies have frequently ignored the benefits of exercise on HTN and all-cause mortality in favor of studying its effects on BP. There are still some gaps in the current literature about exercise and HTN. Thus, we compared groups of non-institutionalized U.S. adults with HTN who were physically active with those who were not, using data from the National Health and Nutrition Examination Survey (NHANES) from 1999 to 2018 to determine the effects of PA on systolic and diastolic BP, as well as all-cause and hypertensive mortality.

## 2. Materials and methods

### 2.1. Study design and population

The data used in this investigation was obtained from 10 rounds of the NHANES from 1999 to 2018. Participants were adults (65 and above) who self-reported a history of HTN, and the study targeted cross-sectional and multi-ethnic groups. Information gathered included self-reported demographic and socioeconomic details and data from medical exams, laboratory tests, and physiological measurements taken and documented by doctors. Everyone who participated in the study had to sign an informed consent form.

### 2.2. Data collection

The NHANES included 11,258 adults who self-reported having HTN between 1999 and 2018. Based on their levels of PA, the participants were divided into 2 groups: 1 that was physically active (at least 30 minutes of PA per session, 5 days a week) and another that was physically inactive (<30 minutes of PA per session, 5 days a week). The final study counted 9525 physically inactive people and 1733 physically active people. During in-depth home interviews with each group, we documented thorough information using questionnaires, including age, gender, race/ethnicity, yearly household income, educational background, current health insurance status, and smoking. Other measurements and records included body mass index, SBP, and DBP. All blood lipid levels and laboratory testing were conducted according to NHANES guidelines. While participating in the study, people taking prescription medications were asked to bring the original containers of all the medications they used. When investigators did not have access to containers, they documented the names of the substances reported orally.

### 2.3. Definition of co-morbidities

Metabolic syndrome was identified as meeting 3 or more of the following criteria^[24]^: (1) SBP > 130 mm Hg or DBP > 85 mm Hg, or receiving treatment for HTN, (2) fasting blood glucose levels between 100 and 125 mg/dL (5.55–6.99 mmol/L) were considered indicative of fasting blood glucose disorder, (3) waist circumference > 88 cm for women or > 102 cm for men, (4) fasting triglyceride > 150 mg/dL (1.7 mmol/L). A waist measurement of more than 102 cm for men and 88 cm for women was used to identify central obesity. In cases when angina pectoris, myocardial infarction (heart attack), or a physician’s diagnosis of coronary heart disease occurred, the condition was deemed to be present. The presence of new symptoms of vasogenic focal neuropathy that continue more than 24 hours and are corroborated by imaging studies like MRI or CT is what medical professionals call a stroke. Heart failure: “Have you ever been diagnosed with heart failure by a health professional?” Answer this question in the affirmative. The presence of diabetes mellitus was determined by whether any of the following conditions were fulfilled: (1) a blood glucose level of 200 mg/dL or higher when not fasting, (2) a fasting blood glucose level of 126 mg/dL or higher, (3) a HbA1c of 6.5% or higher,^[25]^ (4) being treated with hypoglycemic drugs at the moment, and (5) a physician’s diagnosis of diabetes mellitus. When the glomerular filtration rate was <60 mL/min/1.73 m^2^, it was deemed chronic renal disease. The following equation was used to determine the glomerular filtration rate: 186 multiplied by serum creatinine minus 10,154 multiplied by age minus 0.2.3, and then multiplied by (1.212 if Black) and (0.742 if female) according to the modification of diet in renal disease.

### 2.4. Physical-active, BP control, all-cause mortality and hypertensive mortality

The definition of physical-active was to perform it for at least 30 minutes each time, 5 days a week. Control of BP includes BP <140/90 mm Hg, and for individuals with diabetes mellitus or chronic renal disease, <130/80 mm Hg. In each given period, the all-cause mortality rate is the number of fatalities as a proportion of the total population. Conversely, the hypertensive mortality rate is the number of deaths caused by HTN as a proportion of the total population for each period.

### 2.5. Statistical analysis

We used Chi-square tests to look at the percentages in the physical-active group than those in the physical-inactive group by gender, race, socioeconomic class, education level, smoking status, body mass index, and health insurance availability. For this reason, we compared the BP and cholesterol levels of the active and inactive groups using a Student *t* test. We assessed the frequency of co-morbidities in older individuals with HTN using chi-square testing. Diabetes mellitus, central obesity, metabolic syndrome, coronary heart disease, stroke, heart failure, and chronic kidney disease were among the co-morbidities. The distribution of HTN drugs, including β-blockers, α-blockers, diuretics, calcium channel blockers, and angiotensin-converting enzyme inhibitors/angiotensin receptor blockers, was also examined using Chi-square testing. The association between PA and SBP and DBP was further investigated using multivariate linear regression. Various subgroups were investigated using multilevel multivariate regression analysis. These factors encompassed socioeconomic status, racial identity, educational attainment, health insurance coverage, body mass index, and the presence or absence of co-morbidities. Statistical analyses were carried out at a level of significance *P* < .05. For data administration and analytics, we utilized SAS (SAS Institute, Cary, NC, version 9.3).

## 3. Results

Table 1 presents the studied sample, which consisted of 11,258 respondents. With a nearly equal gender distribution, the average age of the participants was 74.36 ± 5.88 years. Non-Hispanic Whites made up around 3-quarters of the respondents, and health insurance was available to most of them. Less than half had earned a bachelor’s degree or above, and about half had a family income of less than $35,000. No statistically significant difference was observed in the prevalence of coronary heart disease between the physically active and physically inactive groups, however, the former had fewer co-morbidities. The physically active group also had lower SBP and used calcium channel blockers and diuretics at a lower rate. Throughout the follow-up period, both the overall and HTN mortality rates decreased.

**Table 1:** Characteristics of the final analytic sample of participants (n = 11,258).

Characteristics	Overall (n* = *11,258)	Physically activity		*P*-value
		Physically active (n* *= 1733)	Physically inactive (n* *= 9525)	
Age (yrs), mean ± SD	74.36 ± 5.88	73.02 ± 5.38	74.60 ± 5.93	<.0001
*Gender, n (%*)				<.0001
Male	5332 (47.36)	1025 (59.15)	4307 (45.22)	
Female	5926 (52.64)	708 (40.85)	5218 (54.78)	
*Race, n (%*)				.06
Non-Hispanic White	6387 (60.16)	980 (62.70)	5407 (59.73)	
Hispanic	2030 (19.12)	288 (18.43)	1742 (19.24)	
Non-Hispanic Black	2199 (20.71)	295 (18.87)	1904 (21.03)	
*Socioeconomic status, n (%*)				<.0001
Low	3008 (50.97)	636 (45.362)	2372 (52.72)	
Middle	1996 (33.82)	512 (36.52)	1484 (36.52)	
High	897 (15.20)	254 (18.12)	643 (14.29)	
*Education status, n (%*)				<.0001
<High school	3948 (35.22)	464 (26.85)	3484 (36.75)	
High school diploma	2737 (24.42)	412 (23.84)	2325 (24.53)	
AA or high	4523 (40.36)	852 (49.31)	3671 (38.72)	
*Current health insurance status, n (%*)				.03
Uninsured	151 (2.04)	42 (2.75)	109 (1.85)	
Insured	7267 (97.96)	1487 (97.25)	5780 (98.15)	
*Smoking, n (%*)				.23
Never smoker	5388 (47.86)	821 (47.37)	4567 (47.95)	
Current smoker	1200 (10.66)	168 (9.69)	1032 (10.83)	
Post-smoker	4670 (41.48)	744 (42.93)	3926 (41.22)	
*Body mass index, n (%*)				.0004
Normal	3633 (35.92)	523 (31.95)	3110 (36.68)	
Overweight	3933 (38.88)	654 (39.95)	3279 (38.68)	
Obesity	2549 (25.20)	460 (28.10)	2089 (24.64)	
*Lipids (mg/dL), mean ± SD*				
HDL-C	54.49 ± 16.82	55.16 ± 17.04	54.31 ± 16.76	.10
TC	187.63 ± 42.90	185.80 ± 40.66	195.05 ± 43.13	.07
LDL-C	106.53 ± 36.07	104.13 ± 34.15	107.15 ± 36.54	.07
*Blood pressure (mm Hg), mean ± SD*				
SBP	143.60 ± 22.29	141.53 ± 20.86	144.00 ± 22.53	<.0001
DBP	66.36 ± 16.59	67.64 ± 14.55	66.12 ± 16.94	.007
SBP*	135.60 ± 21.25	137.48 ± 21.10	140.12 ± 22.70	.0002
DBP*	70.30 ± 15.75	66.53 ± 14.36	64.94 ± 16.56	.002
SBP†	136.57 ± 22.19	146.35 ± 21.35	144.86 ± 25.24	.56
DBP†	77.34 ± 15.38	69.78 ± 15.60	69.78 ± 17.42	.98
*Antihypertensive drugs, n (%*)				
β-blockers	3601 (31.99)	546 (31.51)	3055 (32.07)	.64
α-blockers	548 (4.87)	69 (3.98)	479 (5.03)	.06
Diuretics	3054 (27.13)	403 (23.25)	2651 (27.83)	<.0001
CCB	3077 (27.33)	407 (23.49)	26.70 (28.03)	<.0001
ACEIs/ARBs	5384 (47.82)	875 (50.49)	4509 (47.34)	.02
*Co-morbidities, n (%*)				
MS	1788 (63.18)	315 (54.88)	1473 (65.29)	<.0001
Central obesity	6569 (68.11)	969 (60.68)	5600 (69.58)	<.0001
CHD	2368 (21.03)	339 (19.56)	2029 (21.30)	.09
Stroke	1242 (11.03)	126 (7.27)	1116 (11.72)	<.0001
HF	1090 (9.68)	120 (6.92)	970 (10.18)	<.0001
DM	3080 (27.36)	427 (24.64)	2653 (27.85)	.0054
CKD	1890 (30.77)	291 (23.39)	1599 (32.64)	<.0001
*Mortality, n (%*)				
All-cause	5450 (48.41)	468 (27.01)	4982 (52.30)	<.0001
Hypertensive- cause	1029 (9.14)	55 (3.17)	974 (10.23)	<.0001

Data are presented as n (%) or mean ± standard deviation (SD).

Physical-active: ≥5 days/week and ≥ 30 minutes/session.

Socioeconomic status: low, <$35,000; middle, $35,000 to $75,000; high, >$75,000.

Body mass index (BMI): normal, 18.5 < BMI ≤ 25; overweight, 25 < BMI ≤ 30; obesity, BMI > 30.

Central obesity: waist circumference > 102 cm for men or > 88 cm for women.

AA = associate degree; ACEIs/ARBs = angiotensin-converting enzyme inhibitors/angiotensin receptor blockers; BMI = body mass index; CCB = calcium channel blocker; CHD = coronary heart disease; CKD = chronic kidney disease; DBP = diastolic blood pressure; DM = diabetes mellitus; HDL-C = high-density lipoprotein; HF = heart failure; LDL-C = low-density lipoprotein cholesterol; MS = metabolic syndrome; SBP = systolic blood pressure; TC = total cholesterol.

*P*-value indicates comparison of means or proportions among 2 groups.

* SBP and DBP: the blood pressure for those taking antihypertensive drugs.

† SBP and DBP: the blood pressure for those taking no any antihypertensive drug.

The results of the multivariate linear regression study showed that PA was positively correlated with DBP and negatively correlated with SBP. Table 2 indicates that the association between PA and DBP was not statistically significant when controlling for age, sex, and race in multivariate linear regression models. Even when dividing the patients into 2 groups based on whether they were taking antihypertensive medication or not, no statistically significant relationship was found between PA and BP. Table 3 depicts no statistically significant association between PA and BP control, even after controlling for other variables.

**Table 2:** Coefficients from multiple linear regression of systolic and diastolic blood pressure on physical.

Physical activity level	Systolic blood pressure									Diastolic blood pressure					
	Unadjusted					Adjusted†				Unadjusted			Adjusted†		
	Coefficient		Standard error	*P*-value*		Coefficient		Standard error	*P*-value*	Coefficient	Standard error	*P*-value*	Coefficient	Standard error	*P*-value*
Physical-inactive (active)	2.46		0.60	<.0001		1.30		0.37	.006	-1.52	0.45	.0007	-0.47	0.46	.31
	Systolic blood pressure (taking antihypertensive drugs)									Diastolic blood pressure (taking antihypertensive drugs)					
Physical-inactive (active)	2.64	0.71			.0002	0.74	0.74		.32	-1.58	0.52	.002	-0.80	0.53	.13
	Systolic blood pressure (taking no any antihypertensive drug)									Diastolic blood pressure (taking no any antihypertensive drug)					
Physical-inactive (active)	-1.49	2.85			.60	-2.71	2.94		.36	0.05	1.99	.98	0.74	2.00	.71

Physically active: ≥5 days/week and ≥30 minutes/session.

† Adjusted: were adjusted for age, gender, race.

* Significant *P*-values at 95% confidence interval.

**Table 3:** Physical activity and risks for blood pressure control in hypertensive patients.

Characteristics	Physical activity		*P*-value
	Physically active (n = 4703)	Physically inactive (n = 20,464)	
*Blood pressure control*			
Event, n (%)	748 (43.16)	4018 (42.18)	.45
Unadjusted HR (95% CI)	References	0.96 (0.86–1.07)	.44
Adjusted HR (95% CI)*	References	0.98 (0.88–1.10)	.75
Adjusted HR (95% CI)†	References	1.04 (0.78–1.38)	.79

Physical active: ≥5 days/week and ≥30 minutes/session.

Blood pressure control: blood pressure < 140/90 mm Hg or < 130/80 mm Hg if diabetes or CKD.

CI = confidence interval; HR = hazard ratio.

* Adjusted for age, gender, and race.

† Adjusted for gender, race, socioeconomic status, education status, current health insurance status, low-density lipoprotein cholesterol, high-density lipoprotein, total cholesterol, triacylglycerol, smoking, systolic blood pressure, diastolic blood pressure, body mass index, antihypertensive drugs (β-blockers, α-blockers, diuretics, calcium channel blocker, and angiotensin-converting enzyme inhibitors/angiotensin receptor blockers), and co-morbidities (metabolic syndrome, central obesity, coronary heart disease, chronic heart failure, stroke, diabetes mellitus, and chronic kidney disease).

Table 4 depicts the results of the COX regression analysis. The physical inactivity group showed a substantially increased risk of HTN and all-cause mortality in the uncorrected COX model. After controlling for age, sex, race, and all other factors, the risk of death from any cause and HTN mortality in the physically inactive group remained statistically significant.

**Table 4:** Physical activity and risks for mortality in hypertensive patients.

Characteristics	Physical activity		*P*-value
	Physically active (n = 4703)	Physically inactive (n = 20,464)	
*All-cause mortality*			
Event, n (%)	895 (19.03)	8354 (40.82)	<.0001
Unadjusted HR (95% CI)	References	2.96 (2.65–3.32)	<.0001
Adjusted HR (95% CI)*	References	3.16 (2.80–3.56)	<.0001
Adjusted HR (95% CI)†	References	1.49 (1.08–2.06)	.01
*Hypertensive mortality*			
Event, n (%)	99 (2.11)	1351 (6.60)	<.0001
Unadjusted HR (95% CI)	References	3.48 (2.64–4.58)	<.0001
Adjusted HR (95% CI)*	References	3.29 (2.48–4.37)	<0.0001
Adjusted HR (95% CI)†	References	2.17 (1.04–4.55)	0.04

Physical active: ≥5 days/week and ≥30 minutes/session.

CI = confidence interval, HR = hazard ratio.

* Adjusted for age, gender, and race.

† Adjusted for gender, race, socioeconomic status, education status, current health insurance status, low-density lipoprotein cholesterol, high-density lipoprotein, total cholesterol, triacylglycerol, smoking, systolic blood pressure, diastolic blood pressure, body mass index, antihypertensive drugs (β-blockers, α-blockers, diuretics, calcium channel blocker, and angiotensin-converting enzyme inhibitors/angiotensin receptor blockers), and co-morbidities (metabolic syndrome, central obesity, coronary heart disease, chronic heart failure, stroke, diabetes mellitus, and chronic kidney disease).

The outcomes of the respondent subgroups’ hierarchical multivariate regression analysis are displayed in Table 5. The physically inactive group had a consistently and significantly greater risk of HTN mortality and all-cause death across nearly all subgroups.

**Table 5:** Subgroup analyses of the association between physical activity and the adverse outcomes (all-cause and hypertensive mortalities).

Subgroup	All-cause mortality			Hypertensive mortality		
	Physically inactive vs physically active			Physically inactive vs physically active		
	HR	95% CI	*P*	HR	95% CI	*P*
*Gender*						
Male	2.89	2.50–3.34	<.0001	2.70	1.94–3.74	<.0001
Female	3.53	2.93–4.26	<.0001	5.71	3.34–9.76	<.0001
*Race*						
Non-Hispanic White	2.73	2.37–3.15	<.0001	2.95	2.10–4.14	<.0001
Hispanic	2.82	2.09–3.81	<.0001	3.55	1.80–7.02	.0003
Non-Hispanic Black	4.78	3.45–6.63	<.0001	4.75	2.21–10.19	<.0001
*Socioeconomic status*						
Low	1.64	1.35–1.98	<.0001	2.31	1.47–3.62	<.0003
Middle	2.05	1.60–2.64	<.0001	2.01	1.11–3.66	.02
High	1.56	1.06–2.30	.02	3.53	1.06–11.75	.04
*Education status*						
<High school	3.12	2.53–43.84	<.0001	2.89	1.84–4.53	<.0001
High school diploma	2.78	2.21–3.50	<.0001	3.34	1.89–5.90	<.0001
AA or high	2.73	2.31–3.22	<.0001	3.84	2.46–6.02	<.0001
*Current health insurance status*						
Uninsured	0.37	0.14–0.99	.04	0.38	0.02–6.21	.50
Insured	2.24	1.96–2.56	<.0001	2.96	2.13–4.11	<.0001
*Body mass index, n (%*)						
Normal	2.54	2.05–3.14	<.0001	2.02	1.30–3.12	.0016
Overweight	2.64	2.19–3.18	<.0001	3.73	2.30–6.04	<.0001
Obesity	3.84	3.07–4.79	<.0001	4.98	2.77–8.98	<.0001
*Co-morbidities*						
MS (no)	2.48	1.84–3.34	<.0001	3.07	1.57–6.00	.001
Yes	1.87	1.42–2.45	<.0001	2.48	1.24–4.95	.01
Central obesity (no)	3.69	3.04–4.48	<.0001	4.64	2.82–7.64	<.0001
Yes	2.47	2.12–2.88	<.0001	2.68	1.88–3.82	<.0001
CHD (no)	3.00	2.63–3.42	<.0001	4.23	3.00–5.97	<.0001
Yes	2.90	2.28–3.67	<.0001	2.11	1.32–3.38	.002
Stroke (no)	2.82	2.51–3.18	<.0001	3.77	2.79–5.10	<.0001
Yes	3.91	2.65–5.76	<.0001	1.76	0.87–3.55	.11
HF (no)	2.84	2.53–3.20	<.0001	3.77	2.79–5.10	<.0001
Yes	3.99	2.67–5.95	<.0001	1.76	0.87–3.56	.12
DM (no)	2.83	2.49–3.22	<.0001	3.59	2.61–4.95	<.0001
Yes	3.47	2.74–4.39	<.0001	3.16	1.83–5.47	<.0001
CKD (no)	1.92	1.61–2.29	<.0001	2.61	1.67–4.09	<.0001
Yes	2.10	1.61–2.73	<.0001	2.19	1.25–3.83	.006

Physical active: ≥5 days/week and ≥30 minutes/session.

Socioeconomic status: low, < $35,000; middle, $35,000 to $75,000; high, > $75,000.

Body mass index (BMI): normal, 18.5 < BMI ≤ 25; overweight, 25 < BMI ≤ 30; obesity, BMI > 30.

Central obesity: waist circumference > 102 cm for men or > 88 cm for women.

AA = associate degree; BMI = body mass index; CHD = coronary heart disease; CI = confidence interval; CKD = chronic kidney disease; DBP = diastolic blood pressure; DM = diabetes mellitus; HF = heart failure; HR = hazard ratio; MS = metabolic syndrome; SBP = systolic blood pressure.

## 4. Discussion

This nationwide, multicultural study examined the effects of PA on various variables, including SBP, DBP, all-cause mortality, hypertensive mortality, and others. It involved older persons aged 65 and more with HTN. Based on our findings, we can say that (1) PA was positively associated with DBP and negatively associated with SBP. (2) All-cause and HTN mortality rates were significantly lower in the physically active group compared to the physically inactive group.

Our results are consistent with those of other randomized controlled trials^[26,27]^ and cohort studies^[28,29]^ that have found a negative correlation between PA and BP. In a study of hypertensive older adults, Rondon et al^[30]^ found that BP dropped 22 hours after low-intensity exercise. Furthermore, research has indicated that aerobic exercise for 4 to 12 weeks can reduce approximately 15 mm Hg and resting in resting and around 4 to 9 mm Hg in resting DBP HTN among individuals with essential.^[31]^ A 12-week brisk walking training program significantly reduced SBP immediately after low and high exercise loads, according to a study by He et al,^[20]^ however, DBP did not exhibit any reduction. In a prospective study of 60 individuals with resistant HTN ranging in age from 40 to 75 years, researchers found that aerobic exercise for 12 weeks decreased 24-hour and daytime ambulatory BP and office SBP.^[32]^ Another randomized controlled crossover trial involved nineteen women, showing that high-intensity interval exercise elicited a longer systolic post-exercise hypotension than moderate-intensity continuous exercise compared with the control condition.^[33]^ Dantas et al examined the short-term impact of a time-efficient high-intensity interval training program on ambulatory BP in 21 men with normal BP and reached the same result. A single session of low-volume high-intensity interval exercise in normotensive males decreased average systolic and diastole BP during the awake and 20-hour periods.^[34]^ Water exercise caused post-exercise hypotension (~5 mm Hg) within 21 hours, according to a randomized crossover clinical trial of aquatic exercise carried out by Cunha et al in 24 aged women with HTN.^[35]^ Using ambulatory BP monitoring responses, researchers compared the resting BP of 40 subjects trained in land and aquatic exercises. The results showed that the individuals trained in aquatic exercise had lower SBP and DBP values and that their post-exercise hypotension was faster and lasted longer after aquatic exercise.^[36]^ In addition, functional capacity in elderly HTN patients improved along with BP, metabolic parameters, and physical fitness when exercise training volumes were reduced.^[37]^ However, most of these studies were short-term observations involving small groups of individuals with high BP. In contrast, our study is larger in scale and involves a longer period of clinical observation, which enhances the accuracy of our results.

This study also showed that the rates of death from HTN and all causes are significantly lower among people who regularly engage in PA. These decreases are still statistically significant after controlling for variables such as gender, race, socioeconomic position, level of education, presence or absence of health insurance, blood lipids, smoking, BP, body mass index, drugs, and complications. The positive effects of PA were consistent and statistically significant across all subgroups. Consistent with previous research,^[38]^ our study found that PA lowered the incidence of cardiovascular and all-cause deaths in hypertensive individuals. Xu and colleagues reported^[39]^ that the risk of cardiovascular and all-cause mortality was lowest among individuals with HTN who participated in moderate-to-vigorous PA for at least 7.5 to 15 metabolic equivalent hours per week. A prospective cohort study^[40]^ involving 416,175 individuals found that exercising for an extra 15 minutes daily, on top of the recommended minimum of 15 minutes, further reduced all-cause mortality by 4% and all-cancer mortality by 1%. The study implies that moderate-intensity exercise for either 15 minutes per day or 90 minutes per week might be beneficial. Increasing PA reduced mortality even in patients with stable coronary heart disease.^[41]^ Participation in PA was found to be inversely related to the risk of long-term mortality in both people with and without cardiovascular disease, according to another study.^[42]^ Researchers observed that male survivors of myocardial infarction had a lower risk of mortality if they maintained a high PA or had a long-term rise in PA from before to after the event.^[43]^ Moderate levels of PA during leisure time may be ideal for preventing cardiovascular disease, according to a prospective cohort research in an elderly Chinese population that found that even insufficient activity is linked to a reduced risk of death from any cause of cardiovascular disease.^[44]^ Furthermore, a study that included 34,038 older persons and was comparable to ours found that regular exercise and leisure activities were associated with a lower risk of all-cause death.^[45]^

Nevertheless, there are a few positive aspects to this research. First, compared to earlier research, our estimates are more accurate because of our larger sample size. Second, the NHANES follows a consistent protocol while collecting data, including standardized examinations, data records, interviews, and the inclusion of individuals from a wide range of educational and socioeconomic backgrounds. Third, the NHANES statistics can fairly represent the non-medical institutional population in the United States. Furthermore, there are a few limitations. First, it is difficult to differentiate between main and secondary HTN because our raw data does not reveal the etiology of the disease. Second, recall bias may have skewed the information because the data came from self-reported surveys. Finally, the results may not be generalizable to other racial groups because this study exclusively involved elderly Americans.

## 5. Conclusion

This study showed that PA can reduce SBP in HTN patients aged 65 and above in the US, as well as all-cause and hypertensive mortality, according to data from the NHANES 1999 to 2018. Exercise has substantial non-pharmacological benefits for controlling HTN in the aged, as shown by these results. Thus, it is recommended that aged with HTN engage in physical exercise while guaranteeing their safety since this study’s results support it. Further investigation is required to fully address the physical limitations that prevent aged from engaging in high-intensity exercises, the safety concerns, and the ideal exercise frequency and intensity for BP control.

## Acknowledgments

The authors would like to thank all the staffs of the NHANES and the reviewers who participated in the review, as well as MJEditor (www.mjeditor.com) for providing English editing services during the preparation of this manuscript.

## Author contributions

**Conceptualization:** Lijiang Tang.

**Data curation:** Cheng Xu, Wanqi Yu, Changqing Du.

**Formal analysis:** Cheng Xu, Wanqi Yu.

**Software:** Changqing Du.

**Supervision:** Lijiang Tang, Xiaowei Liu.

**Writing – original draft:** Zhi Zhang.

**Writing – review & editing:** Xiaowei Liu.

## References

[1] Kearney PM Whelton M Reynolds K Muntner P Whelton PK He J. Global burden of hypertension: analysis of worldwide data. Lancet. 2005;365:217–23. 15652604 10.1016/S0140-6736(05)17741-1

[2] James PA Oparil S Carter BL. 2014 evidence-based guideline for the management of high blood pressure in adults: report from the panel members appointed to the Eighth Joint National Committee (JNC 8). JAMA. 2014;311:507–20. 24352797 10.1001/jama.2013.284427

[3] Musini VM Gueyffier F Puil L Salzwedel DM Wright JM. Pharmacotherapy for hypertension in adults aged 18 to 59 years. Cochrane Database Syst Rev. 2017;8:CD008276. 28813123 10.1002/14651858.CD008276.pub2 PMC6483466

[4] Kansagara D Wilt TJ Frost J Qaseem A. Pharmacologic treatment of hypertension in adults aged 60 years or older. Ann Intern Med. 2017;167:291–2. 28806807 10.7326/L17-0286

[5] Maruf FA Salako BL Akinpelu AO. Can aerobic exercise complement antihypertensive drugs to achieve blood pressure control in individuals with essential hypertension? J Cardiovasc Med (Hagerstown). 2014;15:456–62. 24983264 10.2459/JCM.0b013e32836263b2

[6] Benjamin EJ Blaha MJ Chiuve SE. Heart Disease and Stroke Statistics-2017 Update: A Report From the American Heart Association. Circulation. 2017;135:e146–603. 28122885 10.1161/CIR.0000000000000485 PMC5408160

[7] Franklin SS. Hypertension in older people: part 1. J Clin Hypertens (Greenwich). 2006;8:444–9. 16760685 10.1111/j.1524-6175.2006.05113.x PMC8109573

[8] Ciolac EG. Exercise training as a preventive tool for age-related disorders: a brief review. Clinics (Sao Paulo). 2013;68:710–7. 23778419 10.6061/clinics/2013(05)20 PMC3654306

[9] Benjamin EJ Virani SS Callaway CW. Heart disease and stroke statistics-2018 update: a report from the American Heart Association. Circulation. 2018;137:e67–e492. 29386200 10.1161/CIR.0000000000000558

[10] He J Whelton PK. Elevated systolic blood pressure and risk of cardiovascular and renal disease: overview of evidence from observational epidemiologic studies and randomized controlled trials. Am Heart J. 1999;138(3 Pt 2):211–9. 10467215 10.1016/s0002-8703(99)70312-1

[11] Lawes CM Vander Hoorn S Rodgers A. Global burden of blood-pressure-related disease, 2001. Lancet. 2008;371:1513–8. 18456100 10.1016/S0140-6736(08)60655-8

[12] Lewington S Clarke R Qizilbash N Peto R Collins R. Age-specific relevance of usual blood pressure to vascular mortality: a meta-analysis of individual data for one million adults in 61 prospective studies. Lancet. 2002;360:1903–13. 12493255 10.1016/s0140-6736(02)11911-8

[13] Zhou D Xi B Zhao M Wang L Veeranki SP. Uncontrolled hypertension increases risk of all-cause and cardiovascular disease mortality in US adults: the NHANES III Linked Mortality Study. Sci Rep. 2018;8:9418. 29925884 10.1038/s41598-018-27377-2 PMC6010458

[14] Jaeger BC Anstey DE Bress AP. Cardiovascular disease and mortality in adults Aged ≥60 years according to recommendations by the American College of Cardiology/American Heart Association and American College of Physicians/American Academy of Family Physicians. Hypertension. 2019;73:327–34. 30595115 10.1161/HYPERTENSIONAHA.118.12291 PMC6392064

[15] Williamson JD Supiano MA Applegate WB. Intensive vs standard blood pressure control and cardiovascular disease outcomes in adults aged ≥75 years: a randomized clinical trial. JAMA. 2016;315:2673–82. 27195814 10.1001/jama.2016.7050 PMC4988796

[16] Pescatello LS MacDonald HV Lamberti L Johnson BT. Exercise for hypertension: a prescription update integrating existing recommendations with emerging research. Curr Hypertens Rep. 2015;17:87. 26423529 10.1007/s11906-015-0600-y PMC4589552

[17] Pelliccia A Sharma S Gati S. 2020 ESC Guidelines on sports cardiology and exercise in patients with cardiovascular disease. Eur Heart J. 2021;42:17–96. 33180902 10.1093/eurheartj/ehaa735

[18] Oliveira-Dantas FF Browne RAV Oliveira RS Cabral LLP de Farias Junior LF Costa EC. Effect of high-velocity resistance exercise on 24-h blood pressure in hypertensive older women. Int J Sports Med. 2021;42:41–7. 32785911 10.1055/a-1202-1536

[19] Schimitt RP L OC Domingues LB. Effects of a single bout of power exercise training on ambulatory blood pressure in older adults with hypertension: A randomized controlled crossover study. Complement Ther Med. 2020;54:102554. 33183671 10.1016/j.ctim.2020.102554

[20] He LI Wei WR Can Z. Effects of 12-week brisk walking training on exercise blood pressure in elderly patients with essential hypertension: a pilot study. Clin Exp Hypertens. 2018;40:673–9. 29363988 10.1080/10641963.2018.1425416

[21] Oliveira J Mesquita-Bastos J Argel de Melo C Ribeiro F. Postaerobic exercise blood pressure reduction in very old persons with hypertension. J Geriatr Phys Ther. 2016;39:8–13. 25760278 10.1519/JPT.0000000000000049

[22] Marttila J Laitakari J Nupponen R Miilunpalo S Paronen O. The versatile nature of physical activity--on the psychological, behavioural and contextual characteristics of health-related physical activity. Patient Educ Couns. 1998;33(1 Suppl):S29–38. 10889744 10.1016/s0738-3991(98)00007-x

[23] Pescatello LS Franklin BA Fagard R Farquhar WB Kelley GA Ray CA. American College of Sports Medicine position stand. Exercise and hypertension. Med Sci Sports Exerc. 2004;36:533–53. 15076798 10.1249/01.mss.0000115224.88514.3a

[24] McDonagh TA Metra M Adamo M. 2021 ESC Guidelines for the diagnosis and treatment of acute and chronic heart failure. Eur Heart J. 2021;42:3599–726. 34447992 10.1093/eurheartj/ehab368

[25] American Diabetes Association Professional Practice Committee. 2. Classification and Diagnosis of Diabetes: Standards of Medical Care in Diabetes-2022. Diabetes Care. 2022;45(Suppl 1):S17–38. 34964875 10.2337/dc22-S002

[26] Kelley GA Kelley KS. Progressive resistance exercise and resting blood pressure: A meta-analysis of randomized controlled trials. Hypertension. 2000;35:838–43. 10720604 10.1161/01.hyp.35.3.838

[27] Whelton SP Chin A Xin X He J. Effect of aerobic exercise on blood pressure: a meta-analysis of randomized, controlled trials. Ann Intern Med. 2002;136:493–503. 11926784 10.7326/0003-4819-136-7-200204020-00006

[28] Paffenbarger RS Wing AL Hyde RT Jung DL. Physical activity and incidence of hypertension in college alumni. Am J Epidemiol. 1983;117:245–57. 6829553 10.1093/oxfordjournals.aje.a113537

[29] Hu G Barengo NC Tuomilehto J Lakka TA Nissinen A Jousilahti P. Relationship of physical activity and body mass index to the risk of hypertension: a prospective study in Finland. Hypertension. 2004;43:25–30. 14656958 10.1161/01.HYP.0000107400.72456.19

[30] Rondon MUPBO Alves MJNN Braga AMFW. Postexercise blood pressure reduction in elderly hypertensive patients. Cardiopulm Phys Ther J. 2002;13:18. 10.1016/s0735-1097(01)01789-2 11849868

[31] Tabara Y Yuasa T Oshiumi A. Effect of acute and long-term aerobic exercise on arterial stiffness in the elderly. Hypertens Res. 2007;30:895–902. 18049020 10.1291/hypres.30.895

[32] Lopes S Mesquita-Bastos J Garcia C. Effect of exercise training on ambulatory blood pressure among patients with resistant hypertension: a randomized clinical trial. JAMA Cardiol. 2021;6:1317–23. 34347008 10.1001/jamacardio.2021.2735 PMC8340008

[33] Costa EC Kent DE Boreskie KF. Acute effect of high-intensity interval versus moderate-intensity continuous exercise on blood pressure and arterial compliance in middle-aged and older hypertensive women with increased arterial stiffness. J Strength Cond Res. 2020;34:1307–16. 32149879 10.1519/JSC.0000000000003552

[34] Dantas TCB Farias Junior LF Frazão DT. A single session of low-volume high-intensity interval exercise reduces ambulatory blood pressure in normotensive men. J Strength Cond Res. 2017;31:2263–9. 27787467 10.1519/JSC.0000000000001688

[35] Cunha RM Costa AM Silva CNF Póvoa TIR Pescatello LS Lehnen AM. Postexercise hypotension after aquatic exercise in older women with hypertension: a randomized crossover clinical trial. Am J Hypertens. 2018;31:247–52. 28985278 10.1093/ajh/hpx165

[36] Júnior FA Gomes SG da Silva FF. The effects of aquatic and land exercise on resting blood pressure and post-exercise hypotension response in elderly hypertensives. Cardiovasc J Afr. 2020;31:116–22. 31651927 10.5830/CVJA-2019-051 PMC8762840

[37] Moraes WM Souza PR Pinheiro MH Irigoyen MC Medeiros A Koike MK. Exercise training program based on minimum weekly frequencies: effects on blood pressure and physical fitness in elderly hypertensive patients. Rev Bras Fisioter. 2012;16:114–21. 22481693 10.1590/s1413-35552012005000013

[38] Park S Han K Lee S. Cardiovascular or mortality risk of controlled hypertension and importance of physical activity. Heart. 2021;107:1472–9. 33402363 10.1136/heartjnl-2020-318193

[39] Xu JP Zeng RX Lu HN. Analysis of the dose-response effects of physical activity on cardiocerebrovascular and all-cause mortality in hypertension. Front Cardiovasc Med. 2022;9:844680. 35369332 10.3389/fcvm.2022.844680 PMC8969098

[40] Wen CP Wai JP Tsai MK. Minimum amount of physical activity for reduced mortality and extended life expectancy: a prospective cohort study. Lancet. 2011;378:1244–53. 21846575 10.1016/S0140-6736(11)60749-6

[41] Stewart RAH Held C Hadziosmanovic N. Physical activity and mortality in patients with stable coronary heart disease. J Am Coll Cardiol. 2017;70:1689–700. 28958324 10.1016/j.jacc.2017.08.017

[42] Shaked O Cohen G Goshen A Shimony T Shohat T Gerber Y. Physical activity and long-term mortality risk in older adults with and without cardiovascular disease: a nationwide cohort study. Gerontology. 2022;68:529–37. 34515134 10.1159/000518169

[43] Al-Shaar L Li Y Rimm EB. Physical activity and mortality among male survivors of myocardial infarction. Med Sci Sports Exerc. 2020;52:1729–36. 32079915 10.1249/MSS.0000000000002309 PMC7368826

[44] Zhao H Zhang XN Shi Z. Association of level of leisure-time physical activity with risks of all-cause mortality and cardiovascular disease in an elderly Chinese population: a prospective cohort study. J Geriatr Cardiol. 2020;17:628–37. 33224182 10.11909/j.issn.1671-5411.2020.10.003 PMC7657942

[45] Xu L Wang J Li Y. The relationship between physical activity and all-cause mortality among older adults - China, 1998-2018. China CDC Wkly. 2023;5:866–71. 37814611 10.46234/ccdcw2023.165 PMC10560385

